# Editorial: Taking advantage of the JACMP website

**DOI:** 10.1120/jacmp.v9i3.2916

**Published:** 2008-07-31

Readers of the JACMP are well aware that the JACMP is an online journal rather than a print journal. In addition to being less expensive to produce, which enables the JACMP readership to access articles at no cost, an online journal provides the reader with several additional advantages over a print journal.

One advantage is ecological soundness. Electrons are greener than paper. If a reader wishes to have a copy of an article from a print journal, he/she must take the journal to a copy machine and generate a photocopy of the article. One of my professors in graduate school said that “once you have copied an article, you have fulfilled your obligations to the author.” To “fulfill your obligations to the author” of an online journal article, now all you have to do is download the PDF form of the article and store it in a folder on your computer. Only if desired, do you need to generate a hardcopy of the file. Thus the onus of killing trees has been moved from the publisher to the reader.

Other advantages of an online journal exist as well. Viewing an article in its HTML format allows the reader to take advantage of the capabilities of the web. When you view an article on the JACMP website, have a look on the right side of the web page. There you will find additional information about the article and its authors. For example, selecting the “About the author” tab will open up a window that contains brief biographical sketches of the authors, provided such biographical information is available. Authors of the JACMP are encouraged to fill out this information when they submit their manuscripts.

Perhaps the major feature of an article published in HTML is the access to supplementary files. The most familiar type of supplementary files are figures. Clicking on the “Supplementary file” tab, also found on the right side of a JACMP article, will take you to a page of all the figures referenced in an article. Figures that appear in the JACMP can be downloaded and used in other publications and presentations provided proper acknowledgment is given to the author. Copyright of these figures is maintained by the author, who signs a release authorizing distribution of the figures. But please, if you are using an author's figure, be sure to provide proper acknowledgment. Supplementary files may also contain data sets that might be useful for various applications, or they may contain files, such as AVI files of moving entities, that cannot be incorporated into a PDF file of an article. Because of our long tradition with print presentation, we are not used to incorporating dynamic displays in our manuscripts. We encourage the JACMP authors to explore this method for data representation, when appropriate.

Finally, a web‐based journal provides readers an opportunity to comment on articles, much like the reviews of books on the Amazon website (although we don't yet invite readers to rate articles). Good discussion of an article is a welcome addition to any publication. Readers are encouraged to enter their comments onto the JACMP website.

Recently, the issue of plagiarism has come across the desks of online journal editors. Editorials in Medical Physics and Physics in Medicine and Biology have addressed this topic as well, and it is certainly an issue for which we need to have a clearly stated policy. Although the JACMP has not had to deal with plagiarism, it does indeed have a plagiarism policy. A copy of the policy has been placed on our website as a supplementary file to this editorial.

Another current topic is the development of a Bill of Rights for Scientists and Engineers, recently prepared by Bill Hendee, Chair of the International Organization of Medical Physics (IOMP) Publication Committee. Concerns about the intrusion of pressures on the research of scientists and engineers led the president of the IOMP to ask Dr. Hendee to write a Bill of Rights for Scientists and Engineers. This Bill of Rights, which is accessible as a supplementary file to this editorial, responds to the IOMP president's request, and has been approved by the IOMP Council. Because the IOMP is one of the sponsors of the JACMP I have included a copy of the Bill of Rights with this editorial.

As authors and readers of scientific research we are no longer limited by the printed page and as discussed, the advantages of online publishing are numerous. Incorporating features like easy access to supplementary files into our online journals complies with the principles of open access publishing of scholarly resources. I hope that you will expand your articles beyond the printed page and take advantage of all the features that the JACMP website has to offer.

George Starkschall, PhD

Editor‐in‐Chief

August 15, 2008

## Supporting information

Supplementary Material Files

Supplementary Material Files

