# Supplementary material for: Editorial: Taking advantage of the JACMP website
Source: J Appl Clin Med Phys. 2008 Jul 31;9(3):i–ii. doi: 10.1120/jacmp.v9i3.2916 (PMC5722304; doi:10.1120/jacmp.v9i3.2916)
Supplement: Supplementary file 1 — Supplementary Material Files [file ACM2-9-00i-s001.doc]

## JACMP Plagiarism Policy

## The American College of Medical Physics (ACMP) does not tolerate plagiarism or any misrepresentation of original work. In support of this position, the Board of Editors of the ACMP’s Journal of Applied Clinical Medical Physics (JACMP) has adopted the following policies and procedures in response to any accusation of plagiarism.

## Plagiarism is defined as “the appropriation or imitation of the language, ideas, and thoughts of another author, and representation of them as one’s original work.” (The Random House Dictionary of the English Language (unabridged)). Plagiarism is a serious breach of ethics that undermines the authenticity of the JACMP. Plagiarism violates the literary rights of the original author(s) and the property rights of a copyright holder. A person guilty of plagiarism may be subject to legal action instituted by these individuals.

## Upon receipt of a written allegation of plagiarism, the Editor-in-Chief (Editor) of the JACMP shall examine the original material and the publication alleged to constitute plagiarism. If the evidence suggests that plagiarism has not occurred, the Editor shall so notify the accuser and no further action shall be taken. If the evidence suggests that plagiarism may have occurred, the Editor will contact by certified letter the accused author(s), the author(s) whose work may have been plagiarized, and the copyright holder of the original material if different from the author(s). Included in the letter shall be the alleged plagiarizing language, together with a copy of the original and suspected work. If all parties agree that plagiarism (whether intentional or unintentional) has occurred, a written letter of apology shall be sent promptly by the offending author(s) to the Editor and to the author(s) and copyright holder whose work has been plagiarized.

## Upon receipt of an apology, the Editor of the JACMP shall place a Notice of Plagiarism in the journal that contains the offending publication. This Notice shall identify the offending publication, the exact text that has been plagiarized, and the original publication from which the plagiarized text was extracted. Further, the offending author(s) shall agree in writing that no further dissemination of the offending publication shall occur without an attending Notice of Plagiarism.

## If the author accused of plagiarism denies that plagiarism has occurred, the accusation and all supporting materials shall be referred to the Deputy Editor-in-Chief (Deputy) of the JACMP, who shall identify 3 additional members of the Board of Editors to join with him/her in an ad hoc committee to review the accusation and materials. The accused author(s) shall be encouraged by the Deputy to submit any additional information that may be relevant to defense against the accusation. Further, the accused author(s) may request in writing, and be granted, a telephone or personal hearing with the ad hoc committee, with the understanding that expenses of the accused author(s) related to the hearing shall be borne by the author(s). The entire review process shall be concluded in no more than 3 months from the time the author(s) deny the charge of plagiarism.

## If the ad hoc committee rules in support of the accusation of plagiarism, the process described above for the case where plagiarism is admitted shall be instituted. Further, the ad hoc committee shall decide whether the plagiarism is sufficiently egregious to warrant referral to the ACMP’s Ethics Committee, if the person found guilty of plagiarism is an ACMP member.

## If the ad hoc committee rules against the accusation of plagiarism, a letter so stating this ruling shall be provided to the accuser, the author(s) accused of plagiarism, the author(s) of the original work, and the copyright holder if different from the author(s).
